# A Vault Nanoparticle Vaccine Induces Protective Mucosal Immunity

**DOI:** 10.1371/journal.pone.0005409

**Published:** 2009-04-30

**Authors:** Cheryl I. Champion, Valerie A. Kickhoefer, Guangchao Liu, Raymond J. Moniz, Amanda S. Freed, Liisa L. Bergmann, Dana Vaccari, Sujna Raval-Fernandes, Ann M. Chan, Leonard H. Rome, Kathleen A. Kelly

**Affiliations:** 1 Department of Pathology and Laboratory Medicine, David Geffen School of Medicine at UCLA, University of California Los Angeles, Los Angeles, California, United States of America; 2 Department of Biological Chemistry, David Geffen School of Medicine at UCLA, University of California Los Angeles, Los Angeles, California, United States of America; 3 Department of Ophthalmology, University of California Los Angeles, Los Angeles, California, United States of America; 4 Jules Stein Eye Institute, University of California Los Angeles, Los Angeles, California, United States of America; 5 California NanoSystems Institute, University of California Los Angeles, Los Angeles, California, United States of America; New York University School of Medicine, United States of America

## Abstract

**Background:**

Generation of robust cell-mediated immune responses at mucosal surfaces while reducing overall inflammation is a primary goal for vaccination. Here we report the use of a recombinant nanoparticle as a vaccine delivery platform against mucosal infections requiring T cell-mediated immunity for eradication.

**Methodology/Principal Findings:**

We encapsulated an immunogenic protein, the major outer membrane protein (MOMP) of *Chlamydia muridarum*, within hollow, vault nanocapsules (MOMP-vaults) that were engineered to bind IgG for enhanced immunity. Intranasal immunization (i.n) with MOMP-vaults induced anti-chlamydial immunity plus significantly attenuated bacterial burden following challenge infection. Vault immunization induced anti-chlamydial immune responses and inflammasome formation but did not activate toll-like receptors. Moreover, MOMP-vault immunization enhanced microbial eradication without the inflammation usually associated with adjuvants.

**Conclusions/Significance:**

Vault nanoparticles containing immunogenic proteins delivered to the respiratory tract by the i.n. route can act as “smart adjuvants” for inducing protective immunity at distant mucosal surfaces while avoiding destructive inflammation.

## Introduction

Mucosal immune responses provide superior protection against disease but there are currently no FDA-approved adjuvants capable of stimulating cell-mediated immune responses within mucosal tissues [1]. Perhaps one reason is because mucosal immune responses are generated by stimulating mucosal surfaces. However, mucosal surfaces are hostile environments and immunogenic proteins require added protection for delivery to dendritic cells and induction of immunity [2]. We hypothesized that recombinant vaults could provide such protection by encapsulating an antigen and preserving its functional characteristics even within cells [3]. Vaults are ubiquitous, large ribonucleoprotein particles found in nearly all eukaryotic cells including dendritic cells, the endometrium and lung [4]. The vault has a mass of 12.9±1 MDa [5] and a crystal structure at 3.5 Angstrom resolution revealed overall dimensions of approximately 400 Å wide and 700 Å long [6]. Cellular functions of vaults are currently unknown but recent work has found that endogenous vaults may concentrate in lipid rafts and play a role in the uptake of bacteria in the respiratory mucosa [7].

Native vaults are composed of multiple copies of three protein species and several copies of a small untranslated RNA. The most abundant protein, the 97 kDa major vault protein (MVP), is present in 96 copies per vault and forms the barrel-shaped structure of the vault particle [8]. The MVP sequence alone encodes all the needed structural information that enables the protein to self-assemble and form a recombinant vault nanoparticle using the baculovirus system [9]. The internal cavity of the recombinant vault is large enough to accommodate multiple immunogenic proteins. Because vaults are the size of small microbes, a vault particle containing an immunogenic protein should be readily phagocytosed by dendritic cells. Further, recombinant vaults containing proteins are straightforward to produce making vaults a viable vaccine delivery scaffold if they prove facile for generating mucosal immunity.

Mucosal immune responses are optimally produced by stimulating mucosal associated lymphoid tissue. For instance, delivery of immunogenic proteins to nasal surfaces stimulates the induction of immune responses within nasal associated lymphoid tissue (NALT) [10]. Mucosal immune surfaces are interconnected and stimulation of the inductive site in one mucosal surface, produces an immune response at distant mucosal surfaces [11]. In particular, stimulation of the nasal mucosa induces the appearance of immune cells and antibodies in vaginal surfaces [12]. To test the utility of vaults as mucosal vaccine delivery platforms, we chose an infection that relies on cell-mediated mucosal immune responses for elimination and is a significant burden on health care; *Chlamydia trachomatis* infection. Vaginal delivery of the mouse adapted strain of *C. trachomatis*, *C. muridarum*, induces a local sexually transmitted infection (STI) similar to human chlamydial STI [13]. *C. trachomatis* is a prominent cause of STI, with approximately 92 million cases occurring annually [14] and is an instigator of female reproductive dysfunction [15]. T helper immune cells (Th1) must be present within vaginal tissues in order to eradicate infection [16]. However, a vaccine has not yet been produced which induces sizeable Th1 immune responses in mucosal tissues. Therefore, an ideal vaccine would elicit a local anti-chlamydial Th1 cell response in the reproductive mucosa.

## Materials and Methods

### Ethics Statement

All experimental procedures using animals were approved by the UCLA Institutional Animal Care and Use Committee and conducted according to relevant national and international guidelines. All procedures were designed to provide the best possible scientific methodologies available. Since there are no less painful/distressful options available, the techniques have been refined to provide for maximum comfort/minimal stress to the animals. The animals were monitored for pain/distress by exhibiting signs of agitation (licking, biting or guarding the vaginal region), failure to groom, loss of appetite, or marked weight loss (>10%). The Attending Veterinarian was consulted for his/her recommendation for prophylactic treatment when these signs were observed.

### MOMP-mINT Recombinant Vaults

Recombinant baculoviruses were generated using the Bac-to-Bac protocol (Invitrogen, Carlsbad, CA). The 366 amino acid coding region of MOMP (without the signal sequence) was fused to the minimal interaction domain (mINT) derived from VPARP (amino acids 1563–1724) by PCR ligation using the following primers: MOMP-mINT reverse- TCCTGCCAGTGTTGTGTGCAGAAACGGAACTGAGCATTTAC; MOMP-mINT forward- GTAAATGCTCAGTTCCGTTTCTGCACACAACACTGGCAGGA; mINT reverse- GGGCTCGAGTTAGCCTTGACTGTAATGGAG. Two PCR reactions were carried out with the first using a standard T7 primer forward x MOMP-mINT reverse using the MOMP in pcDNA3 as the template. The second PCR reaction used the MOMP-mINT forward x mINT reverse using mINT in pET28 as the template. The PCR reactions were purified on a Qiagen column and a second round of PCR was carried out using the T7 primer forward x mINT reverse. The resultant PCR product containing the fused MOMP-mINT was purified on a Qiagen column, digested with *Bam H1* and *Xho I*, gel purified, and ligated to pFastBac to form a pFastBac vector containing MOMP-mINT. Construction of GL (enhanced green fluorescent protein)-INT in pFastBac has been described previously [3].

Sf9 cells were infected with MOMP-mINT, GL-INT, cp-MVP-z, or cp-MVP recombinant baculoviruses at a multiplicity of infection (MOI) of 0.01 for approximately 65 h and then pelleted and lysed on ice in buffer A [50 mM Tris-HCl (pH 7.4), 75 mM NaCl, and 0.5 mM MgCl2] with 1% Triton X-100, 1 mM dithiothreitol, 0.5 mM PMSF, and protease inhibitor cocktail (2 µg/ml aprotinin, 0.5 µM benzamidine, 2 µg/ml chymostatin, 5 µM leupeptin, 5 µM pepstatin) (Sigma). Lysates containing cp-MVP-z vaults were mixed with lysates containing either MOMP-mINT or GL-INT and were incubated on ice for 30 min to allow the INT fusion proteins to package inside of vaults. Recombinant vaults were purified as previously described [9], [17], [18] and resuspended in 100–200 µl of sterile phosphate buffered saline. The protein concentration was determined using the BCA assay (Pierce) and sample integrity was analyzed by negative stain electron microscopy and SDS-PAGE with Coomassie staining.

### Gel Electrophoresis and Immunoblotting

Sodium dodecyl sulfate-polyacrylamide gel electrophoresis was performed using the discontinuous buffer system and 10–12.5% acrylamide gels. Protein samples of MoPn or MOMP-vaults or whole lysates from dendritic cells were transferred to an Immobilon-P transfer membrane (Millipore) and blocked with 5% (wt/vol) nonfat dry milk in PBS-0.1% Tween 20 (PBS-T). Membranes were individually incubated for 1 h either with antiserum raised against Live CT (1∶500), MOMP-vaults (1∶500), GL-vaults (1∶500), mouse IgG_3_ anti-MoPn-40 (1∶5000), mouse anti-MVP (1∶500, MAB 1023, Santa Cruz Biotechnology Inc, Santa Cruz, CA) or polyclonal rabbit IL-1β (1∶250, sc-7884, Santa Cruz Biotechnology) followed by a 1 h incubation with the appropriate horseradish conjugate (1∶5,000, Amersham Biosciences, Piscataway, NJ). Bound conjugates were detected with SuperSignal West Dura extended duration substrate (Pierce Biotechnology Inc, Thermo Scientific, Rockford, IL) and an Alpha Innotech Fluorchem 8000 imager.

### Anti-vault ELISA Assay

Purified vaults from cp-MVP-z or cp-MVP infected Sf9 cells were diluted to 2 µg/ml in phosphate buffered saline (PBS) and applied to a 96-well dish by serially diluting from 100 ng to 12.5 ng per well (all assays were carried out minimally in duplicate). The plates were incubated at 4°C overnight to allow binding. Unoccupied sites were blocked with 5% BSA in PBS containing 0.05% Tween 20 (PBST) for 1 h at 25°C. After removal of the blocking solution the wells were washed 3 times for 5 min each at 25°C with PBST. Primary antibody (100 µl/well) of either purified mIgG (1 µg/ml, Sigma-Aldrich, St. Louis, MO) or mIgA, kappa-TEPC 15 (2 µg/ml, Sigma-Aldrich) diluted into PBST was applied for 1 h at 25°C. Following removal of the primary antibody the wells were washed as described above. A peroxidase conjugated sheep anti-mouse HRP antibody (1∶1000, GE Healthcare, Piscataway, NJ) in PBST was applied for 1 h at 25°C. After washing the plates, a substrate-chromogen was added (DakoCytomation, Glostrup, Denmark) and developed for 15 min. The reaction was stopped by the addition of 1N H_2_SO_4_. The optical densities were read at 450 nm with a microplate reader (model 550; Bio-Rad, Hercules, CA).

### Dendritic cell Uptake Assay and Flow cytometric analysis

Immature bone marrow dendritic cells (BMDC) from 12 wk old C57BL/6 mice were generated as previously described [19]. The cells were enriched by positive selection using magnetic microbeads conjugated to anti-mouse CD11c mAb (EasySep, STEMCELL Technologies, Inc., Vancouver, BC) and assessed for maturity by measuring expression of MHC class II (anti-IA^b^) and CD40 (eBioscience). Purity of approximately 94% was assessed by flow cytometric analyses acquired on a FACSCalibur flow cytometer with CellQuest Pro software (Becton Dickinson, San Jose, Calif.). BMDC (1×10^6^) were incubated with media, GL-vaults (500 µg) or FITC-dextran (250 µg) (Sigma) for 30–60 minutes at 37°C. These cells were collected, stained with CD11c-PE, a cell surface marker of murine dendritic cells and at least 10,000 cells, based on forward and 90° light scatter properties, were analyzed by flow cytometry. Flow cytometric analysis was performed using FCS Express 2.0 (DeNovo Software, Los Angeles, CA).

### Luminex Suspension Bead Array

Cytokines were measured in the supernates of BMDC or genital tract (GT) homogenates by the Mucosal Immunology Core laboratory at UCLA using the Milliplex Mouse Cytokine/Chemokine Immunoassay (Millipore Corp, Billerica, MA). Multiple, cytokine-specific antibodies were combined to use for the simultaneous measurement of the following mouse cytokines: IL-1α, IL-1β, IL-4, IL-6, IL-10, 1L-12p70, IFN-γ, TNF-α, CCL5 (RANTES), CXCL10 (IP-10) and CXCL2 (MIP-2).

### Toll-like receptor (TLR) Ligand Screening

Empty vaults (cp-MVP-z), GL-vaults, and MOMP-vaults were purified over sucrose gradients by collecting various densities (40, 45 or the interface 40/45) and assessed for potential TLR ligand activity using the InvivoGen TLR Ligand Screening Service (San Diego, CA). TLR stimulation was measured in HEK 293 cells stably transfected with individual plasmids containing mouse TLR 2, 3, 4, 5, 7, and 9 as well as an NF-κB inducible promoter plasmid expressing the secreted embryonic alkaline phosphatase (SEAP) reporter gene. Briefly, 10 µg of each vault prep and positive controls were added to 96-well plates containing 100 µl of reporter cells (5×10^4^/ml). The positive controls employed were TLR2 (heat-killed Listeria monocytogenes, 10^8^ cells/ml), TLR3 (Poly(I:C) 1 µg/ml), TLR4 (Escherichia coli K12 LPS, 100 ng/ml), TLR5 (S. typhimurium flagellin, 1 µg/ml), TLR7 (Loxoribine, 1 mM), and TLR9 (CpG ODN 2006, 1 µg/ml). Cultures were incubated for 24 h and analyzed for NF-κB driven SEAP expression using a substrate followed by absorbance measurements at 650 nm.

### Chlamydia, Immunization and Challenge of mice

*Chlamydia muridarum* (MoPn) was grown on confluent McCoy cell monolayers, purified on Renograffin gradients and stored at −70°C in SPG buffer (sucrose-phosphate-glutamine) as previously described [20]. Female C57BL/6 mice, 5–6 weeks old (Harlan Sprague-Dawley, Indianapolis, IN) were housed according to American Association of Accreditation of Laboratory Animal Care guidelines. Mice receiving vaults were anesthetized with a mixture of 10% ketamine plus 10% xylazine and immunized i.n. with 200 µg MOMP-vaults or 200 µg GL-vaults in 30 µl saline for a total of 3 times every two weeks. As a positive control, a group of mice were immunized i.n. with a single infection of 10^3^ inclusion-forming units (IFU) of MoPn as described [21]. Mice were hormonally synchronized by subcutaneous infection with 2.5 mg of medroxyprogesterone acetate (Depo Provera, Upjohn, Kalamazoo, MI) in 100 µl saline 7 days prior to a vaginal challenge with 1.5×10^5^ IFU of *C. muridarum* and infection was monitored by measuring infection forming units (IFU) from cervical–vaginal swabs (Dacroswab Type 1, Spectrum Labs, Rancho Dominguez, CA) as previously described [20].

### Analysis of T-cell responses

Spleens and iliac lymph nodes (ILN) were harvested from individual mice, dissociated into single-cell suspensions and stimulated for 4 hours with Phorbol myristate acetate (20 ng/ml), ionomycin (500 ng/ml) and Brefeldin A (10 ug/ml) at 37°C, 5% CO_2_. Following incubation, the cells were collected, washed and stained for cell surface markers (CD3,CD4) followed by intracellular cytokine staining (IFNγ, IL-4) and at least 50,000 live cells, based on forward and 90° light scatter properties, were analyzed by flow cytometry as described above. Genital tract (GT) tissue was harvested aseptically from mice at various time-points following intravaginal inoculation with MoPn and divided into cervical-vaginal region (CV), uterine horns (UH), and oviducts (OD) with the ovaries removed as described [22]. The samples were homogenized using a hand-held homogenizer (Omni Intl., Warrenton, VA) on ice in 2 ml of protease inhibitor buffer (Complete Mini Protease Inhibitor Cocktail tablets at a proportion of 1 tablet/25 ml PBS, Roche Diagnostics, Indianapolis, IN). Following homogenation, aliquots were removed to measure chlamydial burden using IFU determination as described [20]. The remaining homogenate samples were centrifuged at 15,000×g for 30 minutes, the supernatants transferred to clean microcentrifuge tubes and stored at 70°C until Luminex analysis was performed.

### Statistics

Two-way repeated measures (RM) analysis of variance (ANOVA) was used to determine statistical differences in the level of infection in the cervical-vaginal swabs (log 10 transformation). Student's t-test or one-way ANOVA was used to evaluate antibody or cytokine levels in tissue homogenates, cell culture supernatants or serum. The above statistical tests were suggested by and performed using SigmaStat software based on the distribution of the data and sample size (Jandel Scientific, San Rafael, CA). Groups were considered statistically different at p<0.05.

## Results

### Design of recombinant vault nanoparticles containing immunogenic proteins

Mucosal immune responses are optimally produced by stimulating mucosal associated lymphoid tissue (MALT). For instance, delivery of immunogenic proteins to nasal surfaces stimulates the induction of immune responses within nasal associated lymphoid tissue (NALT) [10]. Mucosal immune surfaces are interconnected and stimulation of the inductive site in one mucosal surface produces an immune response at distant mucosal surfaces [11]. In particular, stimulation of the nasal mucosa induces the appearance of immune cells and antibodies in vaginal surfaces [12]. We designed vault nanoparticles for use as mucosal immunogens to vaccinate mice.

Vaults are conserved throughout evolution, found in *Trypanosomes* through mammals and are composed of multiple copies of three protein species and several copies of a small untranslated RNA. The most abundant protein, the 97 kDa major vault protein (MVP), is present in multiple copies per vault. Expression of MVP in insect cells using the baculovirus system results in production of vault-like particles formed entirely from the expressed MVP, leading to the conclusion that MVP alone is sufficient to form vault-like particles. These vaults, which lack the two high molecular weight vault proteins (TEP1 and VPARP) and the vault RNA, are less rigid than native vaults [9]. The MVP molecule was modified for increased stability by the addition of a cysteine-rich tag (cp-MVP) [23].

**Figure 1:**
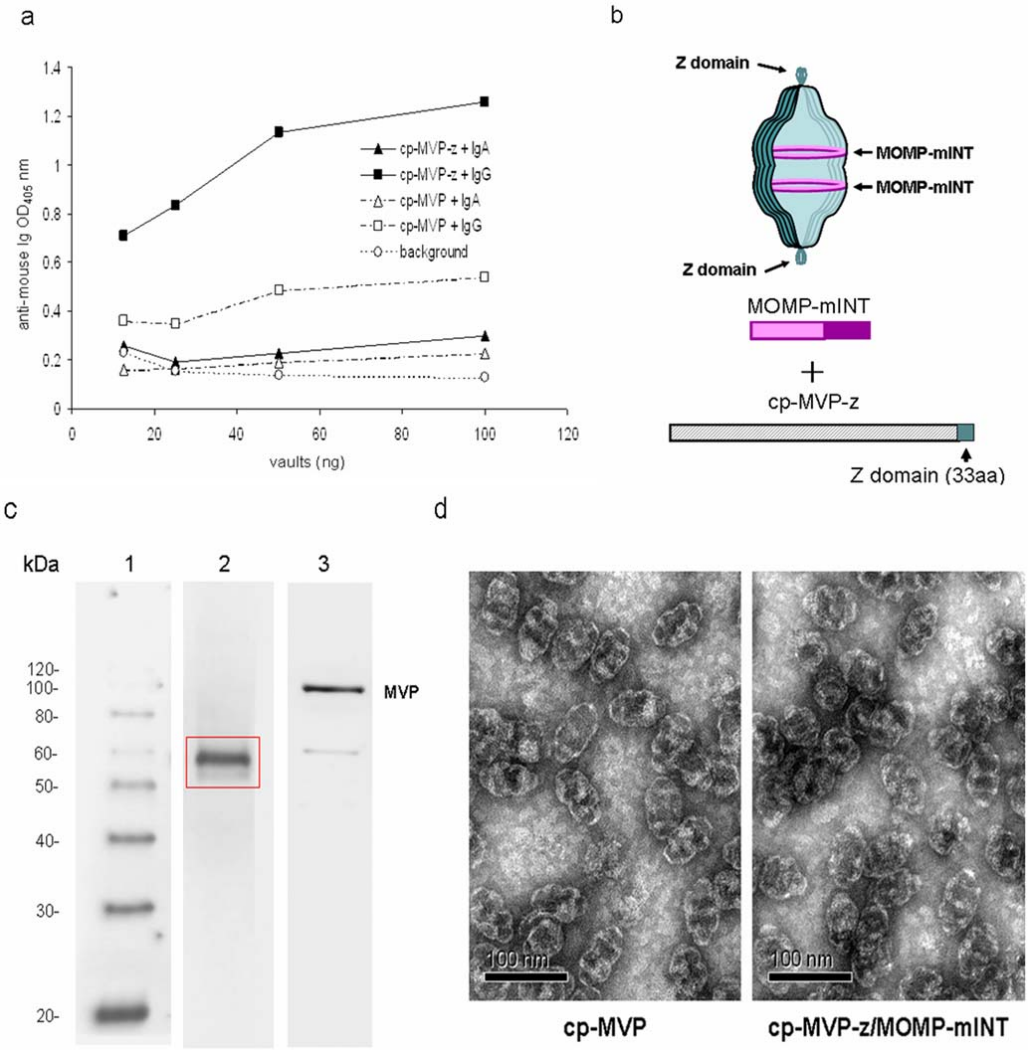
Design of recombinant vault nanoparticles containing immunogenic proteins. (a) ELISA assay configured using vaults with the z peptide (cp-MVP-z) and without (cp-MVP) reacted with either mouse IgG or IgA. Data points represent duplicates, SD = 0.004–0.034 nm. (b) Schematic diagram of constructs used to prepare baculovirus recombinant vaults containing MOMP indicating their approximate locations within a vault. (c) Western blot of high speed pellet extracts of MOMP-vaults (5 µg/lane). Molecular weight markers (lane 1). The gel was probed with a monoclonal antibody against the VD1 region of MOMP (MoPn-40)(lane 2) or mouse 1023C monoclonal antibody against MVP (lane 3). The size of MOMP fused to mINT is shown in the box and is approximately 58 kDa. (d) Negative stain EM of cp-MVP and cp-MVP-z/MOMP-mINT recombinant vaults. Bar, 100 nm.

Delivery of particles to dendritic cells (DC) through the FcR of Ig has been proposed as an effective vaccination strategy for boosting cell-mediated immune responses against *C. muridarum* infection and other pathogens [24], [25]. Further, Th1 responses and immunity against chlamydial genital infection were shown to require the presence of FcR on DC [25]. Cryo-EM and antibody binding studies have shown that molecules on the C terminus of the MVP are displayed on the outside of the particle at the cap [26]. We modified vaults by the addition of a 33 aa peptide (z) derived from staphylococcal protein A to the C-terminus (hereafter referred to as cp-MVP-z) that binds the Fc portion of immunoglobulins at a site distinct from binding to the Fc receptor (FcR) [26]. We tested the ability of vaults expressing (cp-MVP-z) or lacking (cp-MVP) the Fc binding peptide to adhere to mouse Ig isotypes which are generally found in nasal secretions (IgG and IgA) using an ELISA assay. Vaults containing the “z” peptide markedly bound IgG over vaults lacking the “z” peptide. In contrast, regardless of whether or not vaults expressed the “z” peptide, they did not adhere to IgA (Fig. 1a). Thus, the “z” peptide increases binding of mouse IgG to cp-MVP-z vaults.

The internal cavity of the recombinant vault is large enough to accommodate multiple proteins and we packaged the major outer membrane protein (MOMP) of *Chlamydia muridarum* within recombinant vaults. Chlamydial MOMP has immunogenic properties and is able to lessen development of infertility after *Chlamydia* infection [27], [28]. MOMP (without its signal peptide) was fused to the vault-targeting protein, mINT [3], [17] (see Materials and Methods). Purified cp-MVP-z/MOMP-mINT vaults (illustrated schematically in Fig. 1b) were analyzed by Western blot with an antibody specific for the VD1 portion of the *C. muridarium* MOMP verifying that fusion of MOMP to the mINT domain did not alter the ability of antibodies to recognize MOMP (Fig. 1c). Negative stain EM of the cp-MVP-z/MOMP-mINT showed that these vaults were indistinguishable from recombinant cp-MVP vaults (Fig. 1d) and resembled native vaults [29]. Henceforth, we refer to these particles as MOMP-vaults.

### Dendritic cell uptake and maturation by vaults

**Figure 2:**
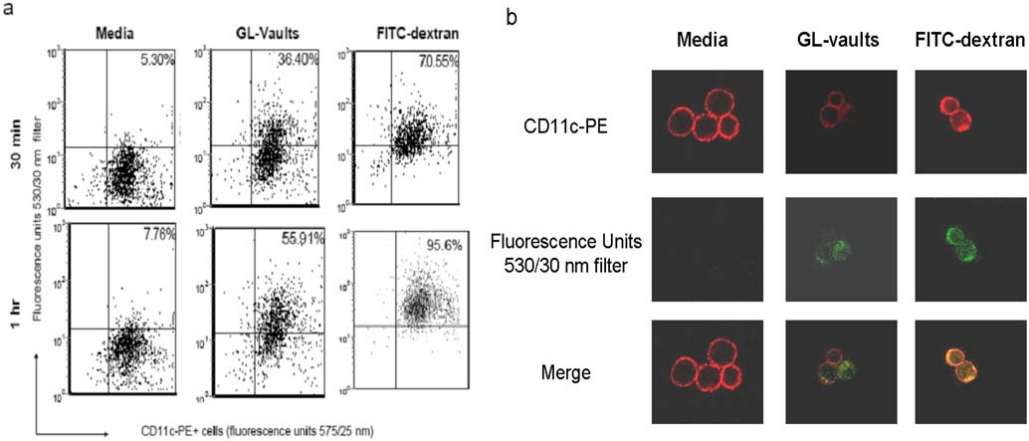
Dendritic cells are efficient at incorporating vaults. (a) BMDC (1×10^6^) were incubated with media, GL-vaults (500 µg) or FITC-dextran (250 µg) at 37°C for the indicated times. Cells were stained for a marker on mouse BMDC (CD11c-PE) and analyzed using a flow cytometer. (b) BMDC (red) incubated as above for 30 min were viewed on a fluorescent microscope (Carl Zeiss, LSM5 Pascal). The fluorescent particles (GL-vaults or FITC-dextran) appear green and mouse BMDC appear red due to staining with CD11c-PE. Results shown are representative of three experiments.

To investigate mechanisms whereby vaults interact with DC to induce immunity, we compared the ability of immature BMDC to incorporate GL-vaults and FITC-dextran using a standard uptake assay [30]. Immature BMDC were produced from C57BL/6 mice [31] and the maturity level measured by lack of cell surface expressed MHC class II and other co-stimulatory molecules by flow cytometry [19]. Immature BMDC (1×10^6^) were incubated with media, GL-vaults or FITC-dextran for 30–60 minutes at 37°C and then stained for CD11c-PE, a cell surface marker of murine DC. GL-vaults incubated with immature CD11c+ BMDC for 30 minutes, resulted in lower levels of fluorescence compared to FITC-dextran. However, at the longer exposure times (1 hr) the rate of uptake was similar between GL-vaults and FITC-dextran (Fig. 2a). A portion of the cells mixed with the green fluorescent particles were fixed on poly-L-lysine coated slides to evaluate particle incorporation. Our analysis showed that GL-vaults (green) were internalized by CD11c+PE+ murine DC (red) and appeared more concentrated in the cytoplasm compared to the FITC-dextran control which mostly adhered to the cell surface (Fig. 2b). This explained the observed kinetics, as FITC-dextran adherence to the cell surface would saturate quickly and appear in the flow cytometry experiments as increased uptake at an earlier time point. Thus, GL-vaults appear to be superior to FITC-dextran for internalization.

**Figure 3:**
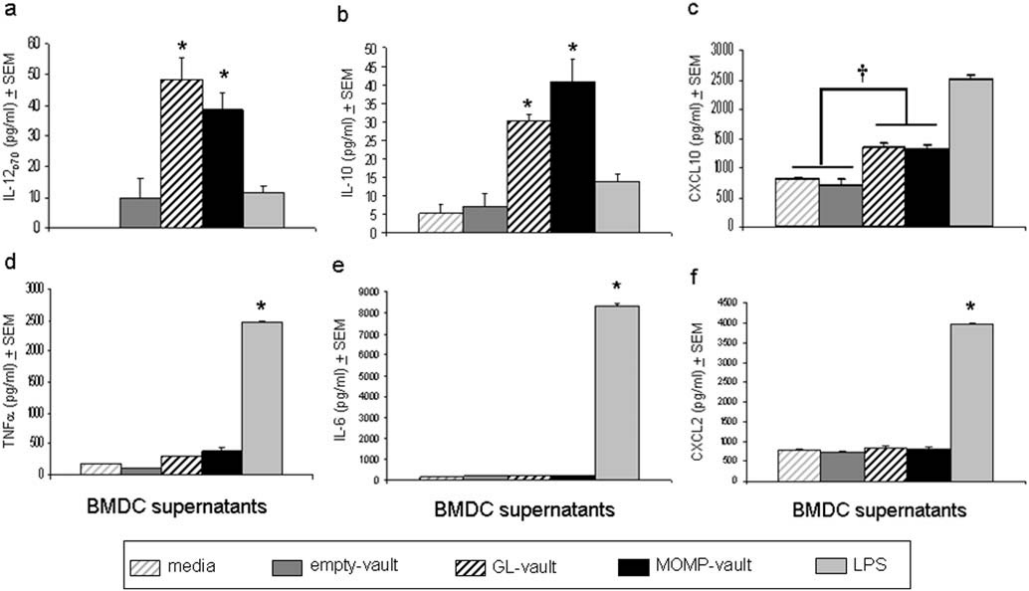
Vaults which contain proteins initiate dendritic cell maturation. BMDC (1×10^6^) were incubated with media alone, empty vaults (500 µg), GL-vaults (500 µg), MOMP-vaults (500 µg) or the control, LPS (100 ng/ml). Culture supernates were collected 18 hrs later and the indicated cytokines, (a) IL-12p70, (b) IL-10, (c) CXCL10, (d) TNFα, (e) IL-6 (e), and (f) CXCL2, were measured by Luminex technology. ANOVA (p<0.001) indicated that the level of cytokines secreted by BMDC differed among conditions. Asterisk denotes a significant (Tukey post-hoc test, p<0.05) elevation in cytokine levels compared to all other conditions. MOMP & GL-vaults significantly increased CXCL10 levels (†; Tukey post-hoc test, p<0.01) compared to media and empty vaults. Cell supernates were measured in triplicate.

Immature BMDC exposed to MOMP-vaults also secreted cytokines and chemokines necessary for producing immune responses. MOMP-vaults significantly increased the secretion of a cytokine needed for production of Th1 cells (IL-12_p70_) compared to empty vaults or the positive control, lipopolysaccharide (LPS) (Fig. 3a). Increases in IL-12_p70_ were balanced by similar increases in IL-10 secretion (Fig. 3b). In addition, MOMP-vaults also significantly increased levels of CXCL10, a chemokine important for attracting Th1 cells (Fig. 3c). Intriguingly, vaults containing another protein, GL, but not empty vaults, also significantly induced the maturation of BMDC by causing secretion of IL-12_p70_, IL-10 and CXCL10 but not IL-4 (data not shown) suggesting that vaults containing proteins favor induction of Th1 immune responses. Unexpectedly, protein-containing vaults did not cause BMDC to secrete the pro-inflammatory cytokines TNFα, IL-6 or CXCL2, a chemokine that attracts neutrophils and a source of tissue inflammation (Fig. 3d–f) indicating that immunization with vaults selectively reduces general inflammation against a specific antigen.

### Vaults do not activate any of the known murine Toll-like receptors (TLR)

**Figure 4:**
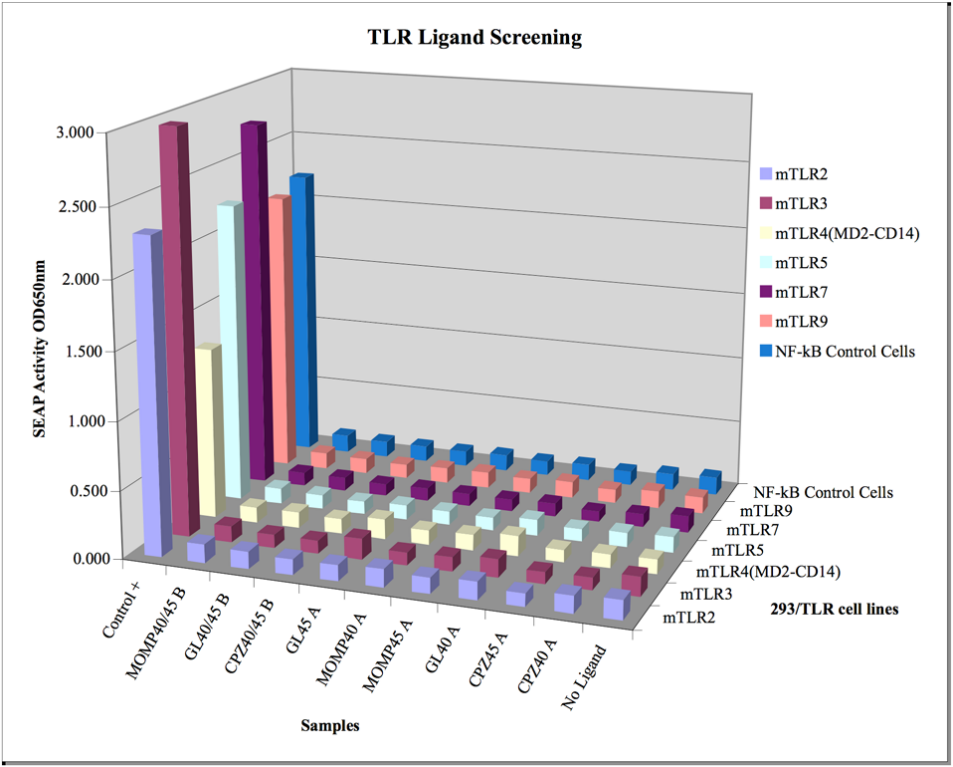
Vaults do not stimulate toll receptors. Vaults were collected from sucrose gradient purification (fraction 40, 45 or a combination) and sent to InvivoGen (San Diego, CA) for analysis of TLR stimulation. At least 10 µg of empty vaults (CPZ), GL-vaults (GL) or MOMP vaults (MOMP) were incubated with NFκB reporter cells (2.5–5.0×10^4^ HEK 293) transfected with a single murine TLR (2, 3, 4, 5, 6, 7 or 9). Controls included TLR2 (heat-killed Listeria monocytogenes, 10^8^ cells/ml), TLR3 (Poly(I:C) 1 µg/ml), TLR4 (Escherichia coli K12 LPS, 100 ng/ml), TLR5 (S. typhimurium flagellin, 1 µg/ml), TLR7 (Loxoribine, 1 mM), and TLR9 (CpG ODN 2006, 1 µg/ml). Secreted alkaline phosphatase (SEAP) activity was measured by absorbance detection. Bars represent the mean absorbance of duplicate wells.

A common cause of cytokine production is ligation of TLR. To determine if any of the known murine TLR are activated by vaults we used an NFκB reporter assay. Various types of vaults (empty, GL or MOMP) were collected from fractions of sucrose gradient purification (fraction 40, 45 or a combination) and sent to InvivoGen (San Diego, CA) for analysis of murine TLR stimulation. Different gradient purified fractions were analyzed for each type of vault. To our surprise, none of the known murine TLR (note that mice do not have TLR8) produced alkaline phosphatase activity, a measure of NFκB activation (Fig. 4). These results indicated that vaults did not induce cytokine secretion from DC through TLR.

### Protein-loaded vaults induce inflammasome activation

Although TLR are responsible for cytokine secretion by many substances they are not responsible for cytokine production induced by alum salts. The adjuvant alum is currently the only FDA approved adjuvant employed in formulations of vaccines for human use. Recently, the mechanism for inducing an adjuvant effect by alum has been identified as inflammasome activation [32], [33]. To date, four different inflammasomes have been characterized [34], containing the Nod-like receptors (NLR): Nalp1, Nalp2, Nalp3 and Ipaf. Alum induces the oligomerization of Nalp3, which in turn causes the recruitment of the adapter protein, ASC (apoptosis-associated specklike protein containing a caspase activation recruitment domain), to form a macromolecular complex called an inflammasome. Two caspase-1 molecules are then recruited and activated by the complex which then leads to the activation of caspase-1 and subsequent IL-1β maturation.

**Figure 5:**
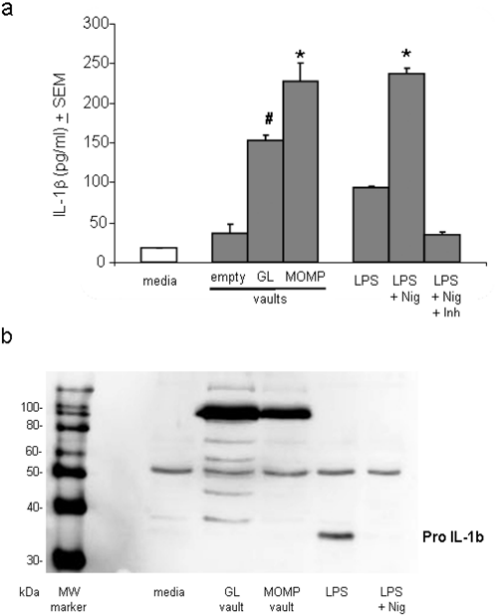
Protein-containing vaults activate inflammasomes. (a) BMDC (1×10^6^) were incubated with the indicated stimulates for 6 hrs (media alone, 500 µg empty, GL-vaults or MOMP-vaults, 100 ng/ml LPS alone or with 3.2 µM nigericin. The caspase-1 innhibitor, 20 µM z-YVAD-fmk, was added to parallel wells containing 100 ng/ml of LPS and 3.2 µM nigericin. IL-1β was measured in triplicate supernatants by Luminex. Bars represent mean IL-1β concentration of triplicates. *p<0.001, compared to all conditions by ANOVA; ^#^p<0.05, Tukey post-hoc analysis comparing the level of IL-1β in supernatants of GL-vault stimulated BMDC to that of supernatants from MOMP-vault stimulated BMDC. (b) Western blot of BMDC cell lysates probed for IL-1β.

We incubated immature BMDC with empty vaults, GL containing or MOMP containing vaults for 6 hrs. Vaults containing proteins but not empty vaults significantly increased IL-1β levels in cell supernatants as measured by Luminex assay (Fig. 5a). LPS by itself is capable of inducing low levels of IL-1β secretion, but IL-1β secretion increased significantly when DC cells were treated with LPS plus the ionophore nigericin (Nig), which activates the Nalp3 inflammasome by inducing K^+^ efflux. These results suggest that MOMP-vaults can both induce pro-IL-1β synthesis and activate the inflammasome, resulting in the maturation and secretion of increased levels IL-1β. This was further confirmed by Western blot analysis showing that pro IL-1β must first be induced by a stimulant, such as LPS, and then cleaved by active caspase-1 produced from activated inflammasomes which causes disappearance of pro IL-1β (p37) in cell lysates from GL, MOMP-vault and LPS+nigericin stimulated DC (Fig. 5b). Note the appearance of a large band with approximate weight of 100 kDa in the lanes containing vaults. This is likely due to the z peptide of cp-MVP-z vaults binding antibody heavy chains in the media. These data, taken together, suggest that cargo-loaded vaults activate inflammasome formation and cause secretion of IL-1β in a TLR-independent manner. Thus, protein-containing vaults caused the maturation of DC which produce cytokines for Th1 differentiation by activating inflammasomes and not TLR. Further, vaults do not cause DC to secrete factors associated with tissue inflammation and are therefore referred to as “smart adjuvants”.

### MOMP-vault intranasal immunization scheme

**Figure 6:**
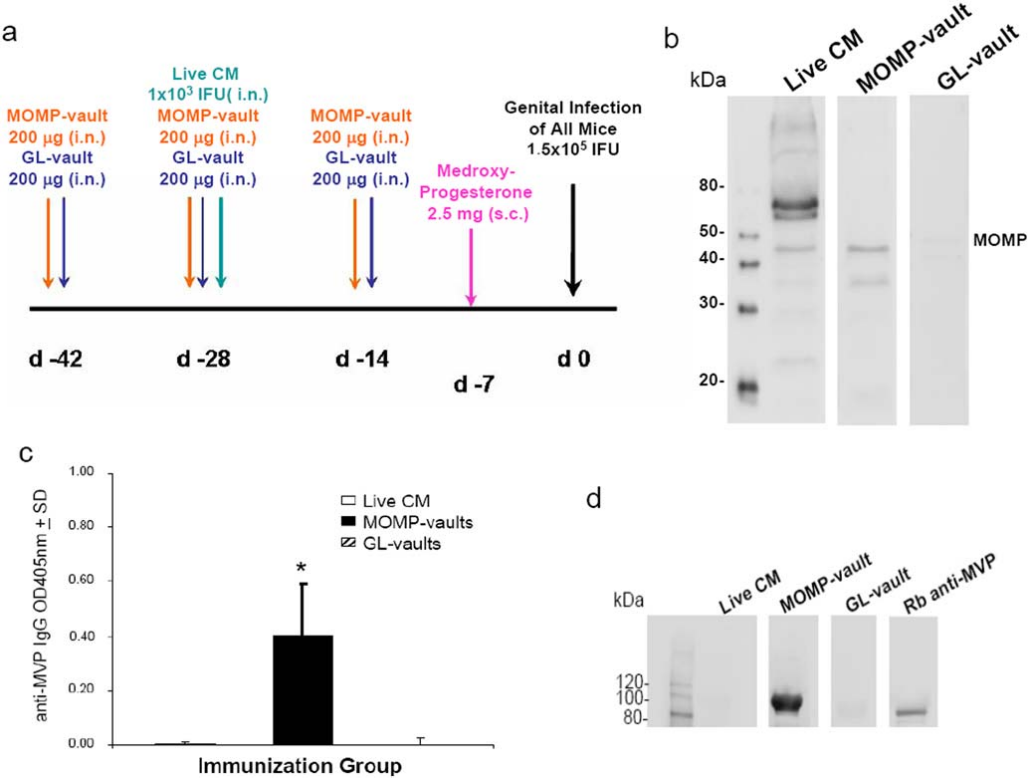
MOMP-vault immunization induces MOMP antibody. (a) C57BL/6 mice were vaccinated according to the indicated schedule and the sera were collected 1 week after medroxyprogesterone acetate injection and right before genital infection. (b) Western blot reactivity of sera pools from the indicated immunization group of mice blotted against a gel containing UV-inactivated, Renograffin gradient-purified chlamydiae elementary bodies. Representative of two independent experiments. (c) ELISA assay of pooled sera against vault-coated plates. Bars represent the mean of 4–6 mice per group and are representative of two independent experiments. * p<0.001, ANOVA. (d) Western blot reactivity of sera pools from the indicated immunization groups or the rabbit monoclonal antibody against MVP blotted against a gel containing MOMP-vaults. Representative of two independent experiments.

To determine if vault “smart adjuvants” containing MOMP could induce mucosal immunity; we used the *C. muridarum* genital infection model. Mice were immunized by delivering the constructed vaults to the nasal mucosa at 2-wk intervals for a total of three immunizations (Fig. 6a). This immunization regimen was shown to reduce infection following intravaginal challenge when soluble *Chlamydia* plus various adjuvants were delivered together [21]. Mice immunized with 200 µg of MOMP-vaults were estimated to contain approximately 0.2 µg MOMP. As a negative control, mice were immunized i.n. with 200 µg GL-vaults [3] and as a positive immunization control (Live-CM), mice were immunized i.n. with 1×10^3^ IFU of chlamydiae [21]. Two weeks following the last vault immunization or 4 weeks after the live lung infection, all mice were hormonally synchronized by subcutaneous injection with medroxyprogesterone acetate to normalize infectivity. All mice were challenged 1-wk later by intravaginal administration with 1.5×10^5^ IFU chlamydiae (Fig. 6a).

### IgG responses after intranasal immunization with vault nanoparticles

Serum antibody responses against chlamydiae were examined to test the vault immunization scheme for development of immunity. Sera were collected from mice 2 weeks after the last immunization, pooled and analyzed by Western blot analysis. As shown in Figure 6b, mice immunized with MOMP-vaults or Live CM produced serum antibody against antigens found on chlamydiae in contrast to mice immunized with the negative control GL-vaults. Mice immunized with MOMP-vaults only produced antibodies that bound to MOMP (MW 45 kDa) or fragments of MOMP compared with the many proteins of chlamydiae that were identified by mice given an i.n. infection with *C. muridarum*. Thus, vaults designed for immunization were capable of producing specific anti-chlamydial IgG.

Vaults are ubiquitous particles found in nearly all eukaryotic cells and would not be expected to induce auto-antibodies [4]. The vast majority (75%) of the vault mass consists of the major vault protein (MVP) [5] and patients with a variety of autoimmune diseases were not found to contain any immune reactivity against MVP (data not shown). However, modifying vault design for increased immunogenicity could possibly break tolerance to this self protein and cause induction of specific antibody. We tested this possibility using ELISA assays on individual sera and found that MOMP-vault immunized mice developed significant antibody to MVP (Fig. 6c). We confirmed this finding in serum pools of mice using Western blot analysis of gels containing cp-MVP vaults. This showed that mice infected i.n. with viable chlamydiae (Live CM) did not produce IgG against vaults when reacted on membranes containing MOMP-vaults (Fig. 6d). Likewise, immune sera from mice immunized with GL-vaults did not produce IgG against MVP (Fig. 6d), demonstrating that repeated i.n. administration of vaults does not induce anti-vault antibody production when designed with the z peptide to enhance development of immune responses. Surprisingly, vaults containing the MOMP protein which is similar in size to the GL-protein, did break tolerance to this self protein and caused the formation of anti-vault antibodies that reacted with the cp-MVP (Fig. 6d). Another fluorescent protein, mCherry, was packaged into a cp-MVP-z vault and used to immunize mice. Sera from these mice also did not produce antibody to cp-MVP vaults by Western blot analysis (data not shown). Thus, development of serum antibody to the vault self-protein is a quality unique to MOMP and not all proteins induce serum antibody formation to MVP.

### MOMP-vault immunization protects against chlamydial genital infection

**Figure 7:**
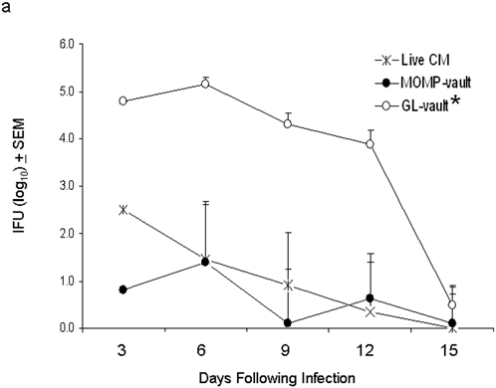
Immunization with MOMP-vaults reduces local bacterial burden following genital infection. The bacterial burden of chlamydiae following a challenge infection was determined from vaginal swabs to be statistically reduced in the MOMP-vault immunized group and the positive control group immunized i.n. with live *C. muridarum* (Live CM) compared to the control GL-vault immunized group (two-way RM ANOVA, *p<0.005). Dunn's post-hoc test showed no difference between the Live CM and the MOMP-vault immunized groups.

The effect of immunization was first evaluated by monitoring the bacterial burden in vaginal swabs collected every 3 days as reported [20].Naïve, non-immune C57BL/6 mice develop an immune response against *C. muridarum* and typically clear a genital infection 2–3-wks later [35] as was observed in mice given GL-vaults (Fig. 7). In contrast, delivery of 200 µg MOMP-vaults significantly reduced IFU levels isolated from vaginal swabs compared to the group immunized with GL-vaults (Fig. 7). More importantly, delivery of MOMP within vaults initially reduced bacterial burden approximately 1 log lower than levels seen in mice naturally immune from a lung infection with live chlamydiae (Live CM) (Fig. 7). This was remarkable protection since in previous studies only MOMP in the native conformation and not recombinant MOMP could achieve this level of protection [36]. Also, a recent study using liposomes containing MOMP in its native conformation as an immunogen followed by vaginal challenge with the same amount of *C. muridarum* (1×10^5^ IFU) did not reach the level of protection seen with MOMP-vault immunization until 10 days after challenge [35]. These data demonstrate that vaults containing MOMP induced a powerful mucosal immunity in the absence of co-delivery with a cytokine [37] or using a live microbial vector [38]. Hence, i.n. delivery of MOMP-vaults was effective at inducing protective immunity as demonstrated by reducing the magnitude of infection upon intravaginal challenge with *C. muridarum*.

### MOMP-vault immunization enhances Th1 cell immunity in mucosal tissues

**Figure 8:**
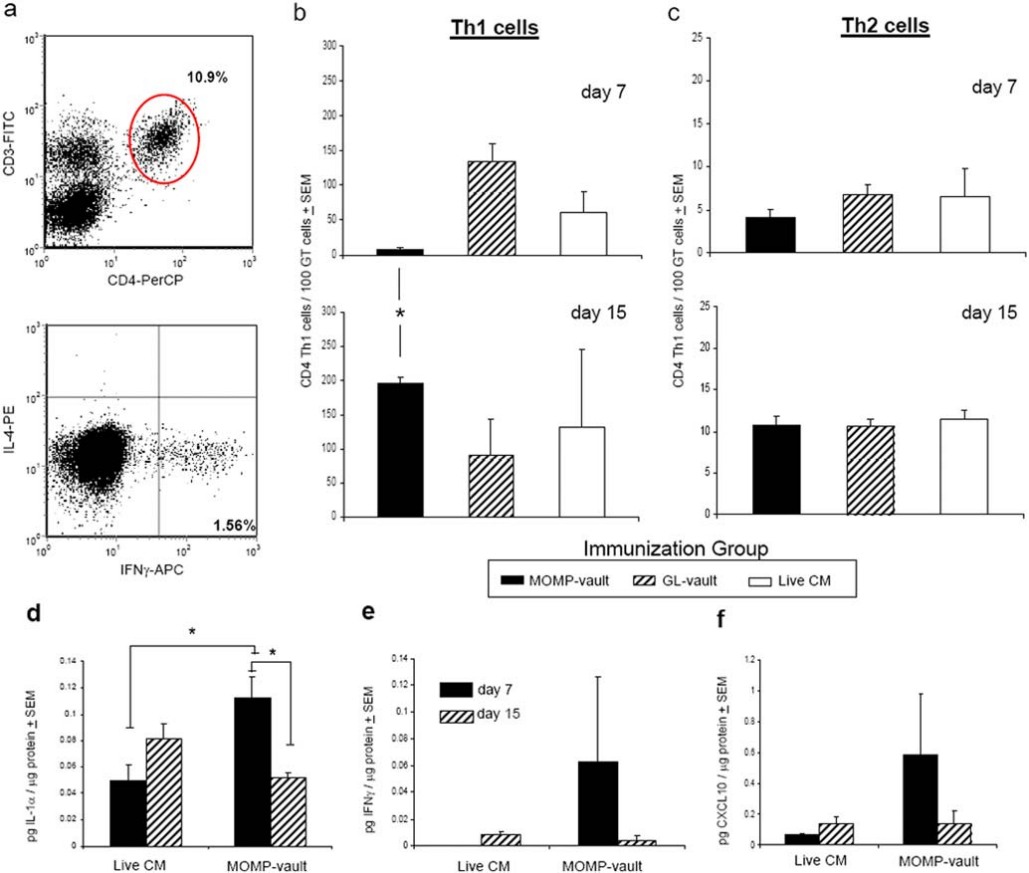
MOMP-vault immunization enhances the number and induces the redistribution of Th1 cells in the ILN. (a) Representative histogram showing CD3+CD4+ cells and IFNγ vs. IL-4 intracellular cytokine staining of ILN cells. The number of (b) Th1 or (c) Th2 cells in the ILNs of individual mice on days 7 and 15 after challenge infection were compared by Students' t-test (*p<0.02). Representative experiment with 4–5 mice per group where at least 50,000 cells were analyzed per mouse. The level of (d) IL-1α, (e) IFNγ, and (f) CXCL10 was measured in triplicate in OD tissue homogenates at two time-points after challenge infection in mice immunized with MOMP-vaults or given a lung infection with *C. muridarum* using Luminex assays (Milipore Corp). Cytokine levels were compared by Students' t-test (*p<0.05). One representative experiment of 3–5 mice per group.

Inflammasome activation facilitates a strong adaptive immune response and promotes the recruitment of immune cells to mucosal tissues [39], [40]. We evaluated Th1 cell immunity in immunized mice after vaginal challenge by quantitating the number of CD3+CD4+ cells that secrete IFNγ or IL-4 (Fig. 8a) in iliac lymph nodes (ILNs) during peak infection (day 7) and during resolution of infection (day 15). MOMP-vault immunized mice produced Th1 cells and demonstrated the migration of Th1 cells by significantly altering the number of Th1 cells found in ILNs at different times after challenge (Fig. 8b). In contrast, mice immunized by a previous lung infection with *C. muridarum* had similar numbers of Th1 cells in ILNs on days 7 and 15. (Fig. 8b). All methods of inducing immunity produced a 10-fold greater number of Th1 cells compared to Th2 cells in the ILNs on days 7 and 15 (Fig. 8c). In addition, we found a significant increase in a cytokine associated with the presence of T cells (IL-1α) in oviduct (OD) homogenates of MOMP-vault immunized mice as early as 7 days after challenge in comparison with immune mice given a lung infection (Fig. 8d). This finding correlated with increased levels of IFNγ in these mice (Fig. 8e). Likewise, we also noted an increase in the level of a chemokine associated with the recruitment of Th1 cells, CXCL10 (Fig. 8f). Taken together, these data indicate that protein-containing vaults activate inflammasomes independently of TLR. Inflammasome-activating agents *in vivo* are capable of producing adaptive mucosal immunity which is characterized in our system by specific antibody production, generation and migration of Th1 cells and protective immunity from a mucosal challenge infection. We hypothesize that protein-containing vaults function as “smart adjuvants” by activating selective properties of DC that stimulate adaptive mucosal immunity. Investigation of the mechanism by which protein containing vaults stimulate mucosal immunity would further development of vaccines.

## Discussion

This study describes the first use of a novel vaccine platform for producing effective cellular immune responses at mucosal surfaces. Intranasal immunization using peptides contained within vault nanoparticles allows development of immunity capable of significantly limiting a bacterial infection in a mucosal tissue. The vault design we employed for immunization was much more effective at reducing bacterial burden following chlamydial genital infection compared to a promising liposome preparation which contains the same antigen (MOMP) and also uses a similar model of chlamydial genital infection [35]. Moreover, immunization with vault nanoparticles did not produce a pro-inflammatory cytokine (TNFα) and a chemokine important for neutrophil recruitment (CXCL2). These results are consistent with the finding that vault nanoparticles did not induce TLR stimulation since TNFα and CXCL2 are produced following TLR stimulation. TLR stimulation is associated with tissue inflammation and not pathogen clearance during chlamydial genital infection [41]. Restraining excess immune responses that are not necessary for eradicating chlamydiae from the genital tract has been proposed to reduce immune-mediated pathology and morbidity following *C. trachomatis* STI [16], [42]. These data indicate that vaults represent a novel adjuvant for inducing protective immunity against microbial infection at mucosal surfaces while limiting excess immunity and investigation of this mechanism will advance vaccine research for mucosal pathogens.

Inflammasome induction is important for producing adaptive immune responses during influenza infection [43] and is responsible for stimulating immune activation with the adjuvant, alum [40]. Moreover, these studies both found that inflammasome formation is required for leukocyte recruitment. Our data show that vault immunization results in the production of factors associated with increased lymphocyte recruitment such as CXCL10 production. Further, vaults induced markedly higher levels of IFNγ, necessary for eradication of chlamydiae, soon after infection in target tissue. Vaults are unique adjuvants that induce activation of inflammasomes but do not stimulate through TLR. Collectively, these results suggest that vaults are superior adjuvants for immunization against infections largely limited to mucosal tissues that rely on leukocyte recruitment for eradication.

The use of recombinant vaults as vaccine platforms to induce immune responses to only the encapsulated protein and not the vault protein appears to depend on the cargo within and not the general use of vaults. Intriguingly, we find that the inclusion of MOMP in vaults was able to break tolerance to this self protein and induce anti-MVP IgG. In contrast, mice exposed to vaults containing other proteins GL or mCherry (data not shown), did not produce antibody to MVP yet could induce maturation of dendritic cells. MOMP has not been reported to induce antibodies that also cross react against host proteins. However, MOMP-vault exposure induced significantly greater levels of IL-1β than GL-vaults indicating that the full length sequence of MOMP within vaults enhances inflammasome formation, possibly as consequence of the porin function or the ability to boost pro-IL-1β. Thus, the degree of inflammasome induction appears to correlate with generation of functional adaptive immune responses. Perhaps development of antibodies to MVP may serve as a screening assay to differentiate the pool of vaccine candidates. The creation of a vault containing appropriate immunogenic peptides that do not break tolerance to self-proteins is the next step for utilizing this effective yet simplistic system for inducing anti-microbial immunity at mucosal surfaces. The ideal vaccine platform would be a “smart adjuvant” that could deliver an antigen to mucosal surfaces and induce rapid anti-microbial immunity while at the same time safeguarding the function of delicate mucosal surfaces by limiting the inflammatory response. Our data indicate that vaults engineered to deliver antigens may in fact act as “smart adjuvants” directing Th1 mediated immunity in mucosal tissues without inducing excessive inflammation.
